# Virtual reality assessment of walking and non-walking space in men and women with virtual reality-based tasks

**DOI:** 10.1371/journal.pone.0204995

**Published:** 2018-10-02

**Authors:** Irene León, Laura Tascón, Juan José Ortells-Pareja, José Manuel Cimadevilla

**Affiliations:** Department of Psychology, University of Almería, Almería, Spain; Universiteit Twente, NETHERLANDS

## Abstract

Far space and near space refer to different spatial features in which we unfold our behaviour. On the one hand, classical visuospatial neuropsychological tests assess spatial abilities in the near space; on the other, far space typically involves new spatial memory tasks in which participants display their behaviour in an environment, either interacting with objects or searching for targets. The Boxes Room Task is a virtual test that assesses spatial memory in the far space. Based upon this task, a new test was developed in which participants could not move about within the context, but they could actually perceive it from a specific viewpoint. In this work, both versions of the task were compared with one another. Furthermore, they were also compared with the results of 10/36 spatial recall test, a task assessing spatial memory in the near space. Two conditions were applied in all tasks, both in stable and rotated contexts. Our study included one hundred and twenty healthy young participants who were divided into two groups. The first group performed the Walking Space Boxes Room Task. A second group performed the Non-Walking Space Boxes Room Task as well as another traditional neuropsychological test for near space assessment, the 10/36 spatial recall test. Results proved that orientation in the non-walking space was more difficult than in the walking space. Additionally, our test also showed that men outperformed women in both virtual reality-based tasks, although they did not do it in the traditional 10/36 spatial recall test. In short, this work exposes that virtual-reality technologies provide tools to assess spatial memory, being more sensitive than traditional tests in the detection of small performance changes.

## Introduction

Spatial orientation is essential in our daily life. Human beings need to remember the position of places and objects around them at all times. Although spatial cognition is the focus of scientists’ attention leading to thousands of experiments in animal models, human spatial memory has been the subject to great attention only in the last few years. Virtual reality-based tasks enable the assessment of spatial orientation in different environments in which participants are demanded to solve a variety of problems [1–4].

It is noticeable that these virtual reality-based tasks provided dissonant data with respect to those data acquired using traditional neuropsychological visuospatial tasks. Hence, new virtual reality based tasks reflect at times a different performance among groups, whereas traditional spatial tasks do not detect such differences [5, 6]. These unmatched results could be explained in connection with the nature of the space in which performance is unfolded. Hence, virtual reality-based tasks usually involve actions within a walking distance (far space), while traditional visuospatial tests require spatial cognition in the short reaching distance (near space). As reported, our brain stores different memories for actions performed in the near and far spaces [7], having involved partially different brain circuits in their retrieval [8].

A completely different issue would be task demands. In many of the virtual reality-based tasks participants can freely explore the environment in a first-person view, adopting viewpoint-dependent strategies. The egocentric spatial reference frame takes special relevance in this modality. Virtual reality techniques have also made it possible to develop tasks in which participants are requested to use an allocentric reference frame. This second approximation would create more difficulties especially if the viewpoint changes in every trial, avoiding egocentric solutions. Obviously, this is relevant at the neuropsychological level since changes on task demands imply that different brain structures underlid performance.

Developed in 2008 [2], the Boxes Room Task was implemented on several segments of the population in the last nine years [2, 5, 6] in which its dependency on the medial temporal lobe integrity was proven [6, 9]. Subjects move about in the virtual scenery by means of a joystick thus enabling the assessment of the walking space. This task demands participants to find one or more rewards in sixteen plausible positions in ten trials. As rewards remained in the same location during the whole experiment, participants could use their previous knowledge to improve across trials. This task will be referred to as Walking Space Boxes Room Task (henceforth WSBRT).

In this study, we developed a version of this task adapted to the non-walking space, which was called Non-Walking Space Boxes Room Task (henceforth NWSBRT). Unlike the original task in which participants had to walk about and explore a virtual room so as to locate the rewarded positions, in the new task subjects used a computer mouse to select the locations in which they considered a reward would be obtained. It is noteworthy that an important difference between walking and non-walking space tasks would allow participants to see the complete set of stimuli in the non-walking space, whereas in the walking space the set of stimuli shown depends on the position of the participant who could move about in specific room locations enabling the implementation of viewpoint-dependent strategies.

In order to avoid egocentric solutions, the starting point is changed on each trial in many of the spatial cognition tasks used in humans and animal models. This procedure is uncommon in traditional visuospatial neuropsychological tasks like 10/36 Spatial Recall Test (10/36 SRT) where the board is kept stable during assessment. A comparison of both conditions, stable vs moved, could help to understand strategies chosen to reach different solutions.

Furthermore, spatial cognition is directly related to gender. Hence, men and women have proven to use different spatial abilities in which male outperform their female counterparts. This was previously reported in several virtual reality-based tasks [1, 10] as well as in different versions of the Boxes Room task [11, 12]. Women committed more errors than men [12] no matter if they used distal landmarks or proximal landmarks. It is noticeable that sexual dimorphism depended on the level of difficulty [4, 10]. Thus, differences appeared in levels of medium difficulty but disappeared when low or high difficulty conditions were used [2, 11,12].

In this manuscript we studied men and women spatial memory using two versions of the Boxes Room Task for walking and non-walking spaces, as well as a traditional visuospatial neuropsychological task, 10/36 SRT. Rotated and fixed conditions were implemented to compare performance in both types of space. We hypothesized that virtual reality-based tasks will be more sensitive to sample conditions than traditional neuropsychological tests. In addition, spatial learning will take longer in allocentric reference frames, having men outperform women in both types of space.

## Materials and methods

### Participants

One hundred and twenty participants were recruited for this study (60 men and 60 women), ages ranging from 18 to 36 years old (Table 1). They all were students majoring in Psychology and Physiotherapy at the University of Almeria, Spain. Exclusion criteria included a history of addictive substances, psychiatric disorders, neurology disease, brain damage or any other condition that could interfere with performance. No student had any previous experience with the type of task in which they were participating. Previous videogame experience was registered using a Likert scale (1, never; 2, rarely; 3, occasionally; 4, frequently playing videogames). Participants were semi-randomly distributed into four groups: WSBRT stable, WSBRT rotated, NWSBRT stable, NWSBRT rotated. Each group consisted of fifteen men and fifteen women. Those subjects assigned to NWSBRT stable and NWSBRT rotated groups performed the stable and rotated versions of 10/36 SRT, respectively.

**Table 1:** Number of participants and age in each test.

	**Males (n = 60)**			
**WSBRT**	**Age**	**Handedness (R/L)**	**Videogame Experience**	**Alcohol Consumption (Y/N)**
Condition 1 (stable)	20.1 ± 2.2	14/1	3.2 ± 0.8	8/7
Condition 2 (rotated)	20.9 ± 3.4	13/2	2.7 ± 0.8	7/8
**NWSBRT/10/36 SRT**				
Condition 1 (stable)	21.2 ± 5.1	13/2	3.1 ± 1.1	9/6
Condition 2 (rotated)	21.4 ± 4.7	15/0	2.6 ± 1.1	7/8
	**Females (n = 60)**			
**WSBRT**	**Age**	**Handedness (R/L)**	**Videogame Experience**	
Condition 1 (stable)	21.5 ± 4.9	13/2	1.9 ± 0.8*	6/9
Condition 2 (rotated)	19.7 ± 0.8	15/0	1.9 ± 0.6*	7/8
**NWSBRT/10/36 SRT**				
Condition 1 (stable)	19.5 ± 1.7	15/0	2.1 ± 0.4*	8/7
Condition 2 (rotated)	20.5 ± 2.2	15/0	2.1 ± 0.5	7/8

* p < 0.05 when compared with the male counterpart (Student’s t-test).

The study was approved by the Ethics Committee of the University of Almeria and conducted in accordance with the European Communities Council Directive 2001/20/EC and the Helsinki Declaration for biomedical research involving humans. All participants were informed in advance about the experiment and they all signed consent forms.

### Apparatus

The virtual spatial memory tasks for walking and non-walking space were administered on a Hewlett Packard 2600-MHz notebook equipped with 3 GB of RAM and 15.4 XGA TFT colour screen (1920 X 1200 pixels). Furthermore, a joystick and a mouse were used in the assessment of walking and non-walking space, respectively. The computer emitted both auditory and visual feedback.

### Procedure

#### Walking Space Boxes Room Task (WSBRT)

**Fig 1:**
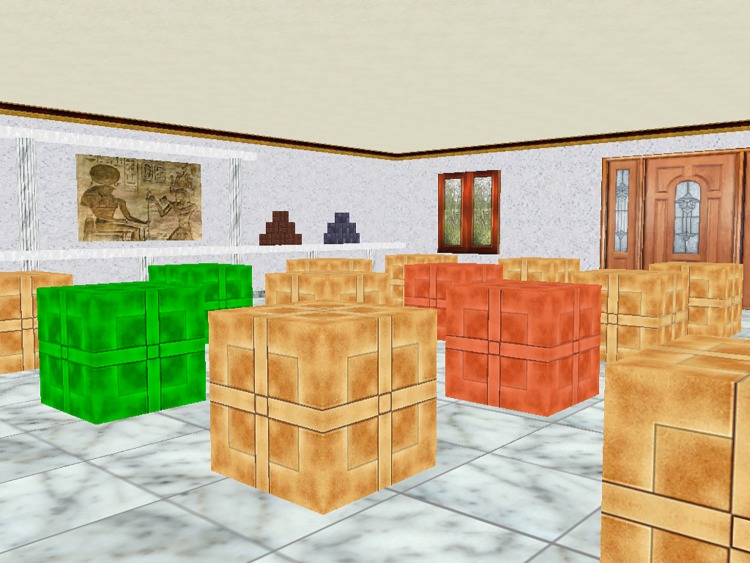
WSBRT. Representative view of the virtual room. Participants have to move into the virtual room to select the rewarded positions. Correct boxes turned green, whereas red indicates wrong boxes. Non-open boxes remained brown. Rewarded positions remained stable during the ten-trial experiment.

The Walking Space Boxes Room Task (WSBRT), as described in Cánovas et al. (2008) [2], consisted of a computer-generated square room with 16 boxes distributed on the floor. A door, a window and several pictures were used to disambiguate spatial locations. Participants were instructed to find reward boxes as quickly as possible by opening the least possible number of boxes. They were also informed about the number of reward boxes and that their positions remained constant during the ten trial experiment, (Fig 1). No information with regard to useful strategies, the location of the rewarded boxes, nor additional features of the experiment were given to subjects.

Provided with a joystick participants were asked to walk about the virtual room to select and open reward boxes. When a reward box was opened, it turned green while a pleasant melody sounded; on the other hand, non-reward boxes turned red followed by an aversive, discordant melody sound. The previously opened boxes remained green or red during the trial session helping participants remember the opened positions, whereas unopened boxes remained brown. As soon as all reward boxes were located, or once 150 seconds had elapsed—the maximum trial duration—a congratulating message popped up on the display screen indicating the participant that the next trial was about to start. Three levels of difficulty were used. All participants were initially tested in a three-reward condition, followed by five and seven-reward conditions. Subjects received ten trials in each condition. The starting position remained stable in the stable condition groups whereas it was changed semi-randomly in the rotating condition groups. Note that rotation prevents egocentric solutions of the task. The number of errors was registered in each trial.

#### Non-Walking Space Boxes Room Task (NWSBRT)

**Fig 2:**
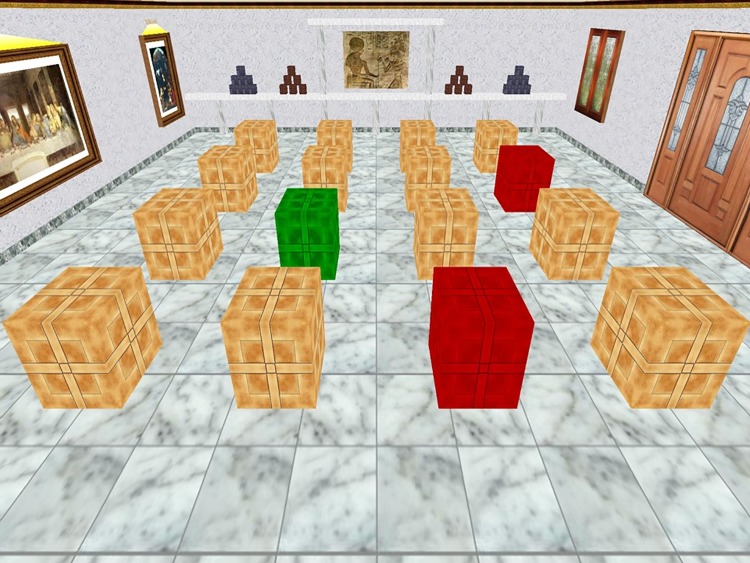
NWSBRT. Representative view of the virtual room. Note that the context is similar to WSBRT. Now, each participant opened the brown boxes with a double-click on the mouse. Green indicated a reward box was opened and red indicated a non-reward one.

The virtual room was adapted from Cánovas *et al*., 2008 [2]. Participants could see the virtual room from one of the four walls. From that point of view, it was possible to appreciate the other three walls with several landmarks (window, door, paintings…) and a total of 16 brown boxes ordered in rows of 4 (Fig 2). By means of a PC mouse, participants had to open the boxes by placing the cursor over them. Once they clicked on the box, its colour changed: it turned to green if the response was correct and red when the response was wrong. A different sound accompanied both correct and incorrect responses. Participants were asked to find the reward boxes (green colour) avoiding the red ones. The trial finished when participants had found all the green boxes or when 150 seconds had elapsed. As in the previous experiment, participants could take advantage of previous trials to improve performance in forthcoming trials, since reward boxes (green boxes) remained in the same position during the whole experiment.

Three levels of difficulty were faced by each participant: in the first one, subjects needed to find three green boxes, whilst in the second condition and the third condition the number of reward boxes increased to five and seven boxes, respectively. For each condition a total of ten trials were presented. Starting positions changed semi-randomly in the rotated condition. Number of errors per trial was registered for each subject.

#### 10/36 Spatial Recall Test (SRT 10/36)

**Fig 3:**
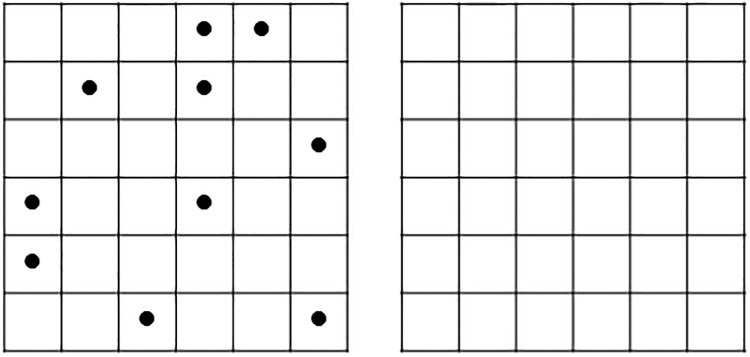
10/36 SRT. On the left side, the template lamina was presented for ten seconds followed by the blank one (right side). Participants were asked to remember the positions occupied by the dots. The original laminas size was 29 x 29 cm.

This traditional neuropsychological test for assessing spatial memory was included in both experiments [13] (Fig 3). It provided information about the performance in the near space (reaching space). Two conditions were used. In the stable condition, the template remained in the same position for all the participants. Conversely, the template rotated 90° on trials 2nd, 3rd and 4th. Participants who performed the stable condition in the NWSBRT received also the stable 10/36 SRT and vice versa.

### Statistical analyses

The number of errors during the different conditions in the WSBRT and NWSBRT were analysed using a five-way ANOVA (Gender x Maze x Condition–stable vs rotated- x Difficulty (3, 5 and 7 rewards) x Trial) with repeated measures in the last two variables. The first trial was excluded from analyses since performance is completely at chance.

Correct responses in the 10/36 SRT were analyzed using a three way ANOVA (Gender x Condition–stable vs rotated- x Trial) with repeated measures in the last variable.

A post-hoc Newman-Keuls test was used when necessary. The statistical analyses were performed using the STATISTICA, 10.0. Differences in which p<0.05 were considered significant.

## Results

A five-way ANOVA (Gender x Maze x Condition x Difficulty x Trial) with repeated measures in Difficulty and Trial disclosed a significant main effect of Gender (F(1,112) = 5.36, p = 0.022), Maze (F(1,112) = 11.98, p = 0.0007), Condition (F(1,112) = 40.7, p = 0.0001) and Trial (F(8,896) = 96.5, p = 0.0001) but no significant effects of Difficulty (F(2, 224) = 1.08, p = 0.34). In addition ANOVA revealed a significant main effect of the interaction terms showed on Table 2.

**Table 2:** Statistically significant interactions.

Factor	F value
Maze x Condition	F(1,112) = 28.06, p = 0.001
Gender x Maze x Condition	F(1,112) = 6.86, p = 0.05
Difficulty x Condition	F(2,224) = 9.42, p = 0.001
Difficulty x Maze x Condition	F(2,224) = 7.16, p = 0.001
Difficulty x Trial x Maze	F(16,1792) = 3.29, p = 0.001
Difficulty x Trial x Condition	F(16,1792) = 2.40, p = 0.001
Difficulty x Trial x Gender x Condition	F(16,1792) = 1.72, p = 0.05
Trial x Maze	F(8,896) = 2.37, p = 0.05
Trial x Condition	F(8,896) = 6.98, p = 0.001
Trial x Maze x Condition	F(8,896) = 2.64, p = 0.05
Trial x Gender	F(8,896) = 2.19, p = 0.05
Trial x Gender x Maze x Condition	F(8,896) = 2.13, p = 0.05

**Fig 4:**
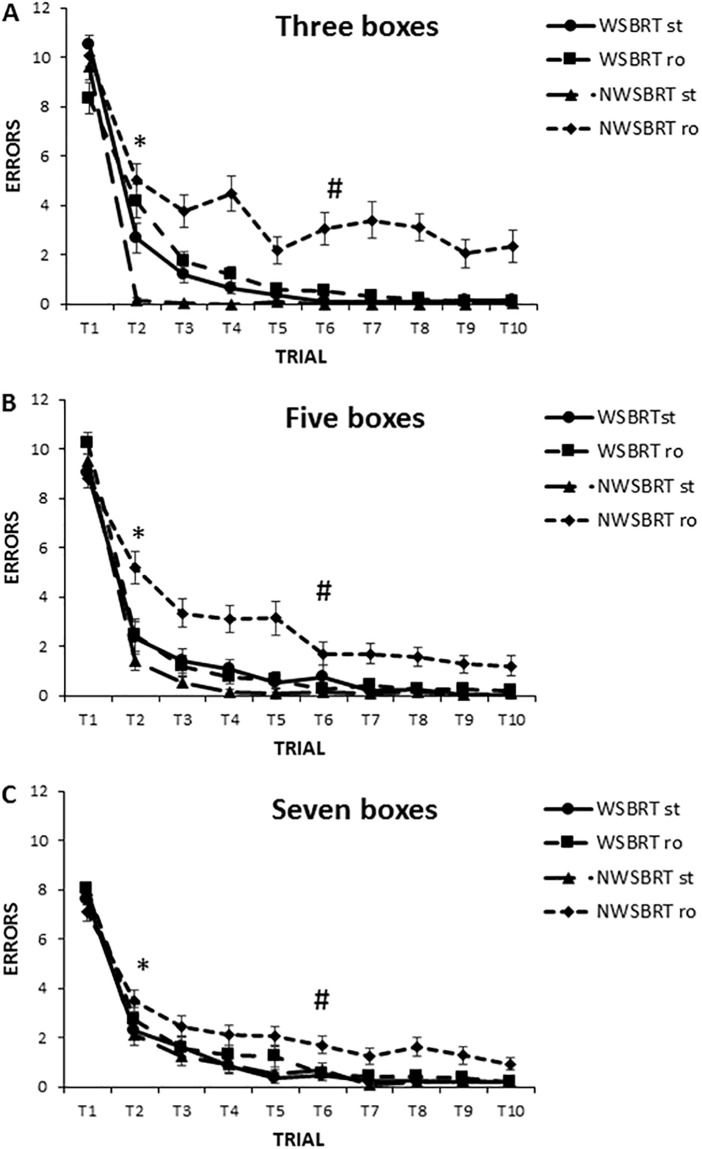
Number of errors in WSBRT and NWSBRT (stable and rotated conditions). A, B and C for three, five and seven boxes conditions, respectively. Note that participants made more errors in the rotated NWSBRT (#). This clearly shows that the rotation of the environment increase the difficulty of the task. The number of errors decreased on trial 2 (*). ST = stable; RO = rotated. Mean±SEM.

A post-hoc analysis of the interaction term Trial x Gender x Maze x Condition disclosed that participants made more errors in the NWSBRT than in the WSBRT and more errors in both tasks in the rotated condition than in the stable one (p<0.05) (Fig 4).

**Fig 5:**
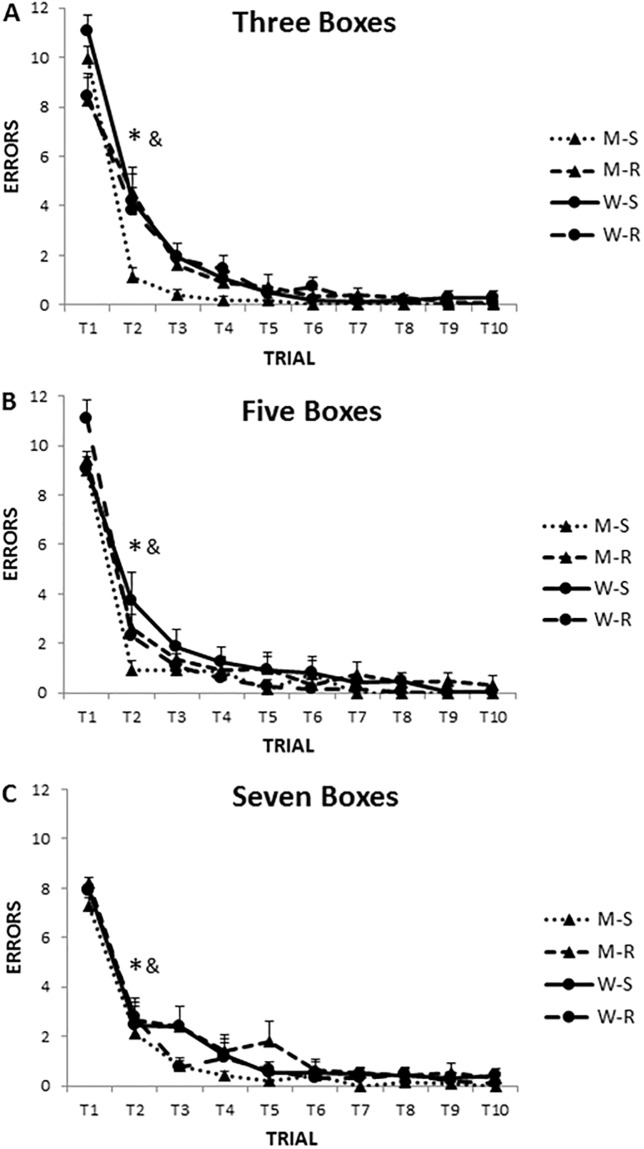
Number of errors in the WSBRT. A) Three boxes condition. B) Five boxes condition. C) Seven boxes condition. In the stable condition, men achieved the asymptotic level sooner than women searching for three and five positions. & gender differences; * decrease of number of errors (p<0,05). M-S (men stable), M-R (men rotated), W-S (women stable), W-R (women rotated). Mean±SEM.

**Fig 6:**
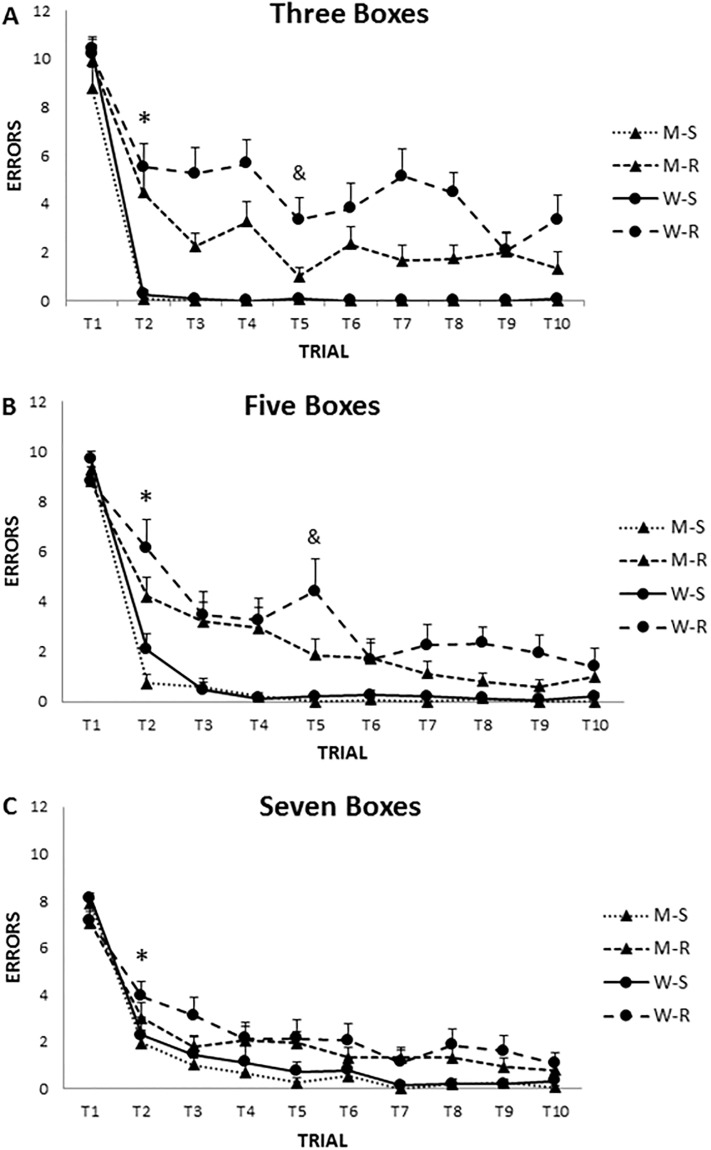
Errors made in the NWSBRT. A) Three boxes condition. B) Five boxes condition. C) Seven boxes condition. Note that in the stable condition, errors increased with task difficulty. Conversely, errors decreased with the increase of the level of difficulty in the rotated condition. & gender differences; * decrease of number of errors (p<0,05). M-S (men stable), M-R (men rotated), W-S (women stable), W-R (women rotated). Mean± SEM.

Analysing the same interaction, Trial x Gender x Maze x Condition, some gender differences emerged. Thus, NWSBRT and WSBRT differed in the rotated condition in men but no differences were found in the stable condition. Nevertheless, NWSBRT and WSBRT differed in both versions (stable and rotated) in women. In addition, men outperformed women in the stable condition of the WSBRT (trial 2) and rotated condition of NWSBRT (trial 5) (p<0.05). Also men achieved the asymptotic level of performance sooner than women in the stable condition in both mazes (trials 2 and 3 vs. trials 3 and 4) for NWSBRT and WSBRT, respectively (p<0.05) (Figs 5 and 6).

The analysis of the interaction term Difficulty x Maze x Condition, revealed that participants decreased the number of errors in the NWSBRT (rotated version) as the number of positions increased (mean = 3.25, 2.46, 1.86 for three, five and seven positions, respectively) (p<0.05).

Moreover, other post-hoc analyses demonstrated that participants made more errors in trial 2 in the WSBRT searching for 3 positions than searching for 5 or 7 positions (p<0.05). This indicates that experience modulates difficulty. In addition, no differences emerged in both mazes between 3, 5 and 7 positions in the stable condition, but in the rotated one errors decrease as the number of positions to be found increased (p<0.05).

### 10/36 Spatial Recall Test

**Fig 7:**
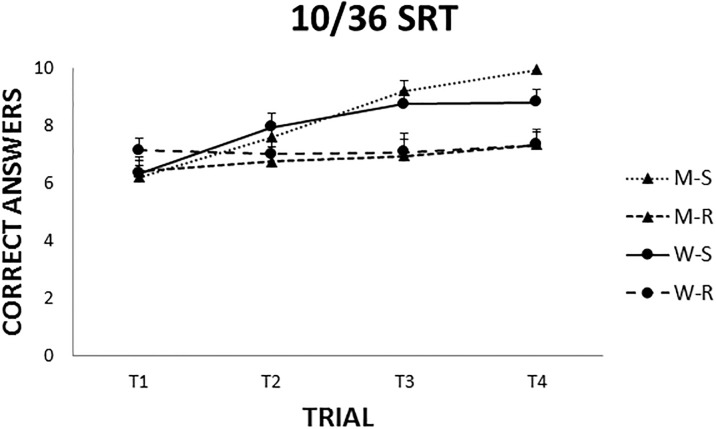
Errors made in the 10/36 SRT. Number of errors is lower when the board is stable along trials. Participants made more errors in the first trial in the stable condition. Mean± SEM.

Participants showed a better performance when the board was stable between trials. A three way ANOVA (Gender x Condition x Trial) with repeated measures in the last factor, revealed a significant main effect of Condition (F(1,56) = 9.06, p = 0.003) and Trial (F(3,168) = 16.09), p = 0.001) but no effect of Gender (F(1,56) = 0.001, p = 0.99). In addition Condition x Trial was also statistically significant (F(3,168) = 8.71, p = 0.001). A post-hoc analysis of the interaction term revealed that participants made more errors in the first trial in the stable condition (always the same board position). It is noteworthy that their performance improved in the third and fourth trials in comparison to the second trial (p<0.05). No differences between trials appeared in the rotated condition, showing that performance did not improve (p>0.05). The number of errors, however, differed between stable and rotated condition in the third and fourth trials, thus participants making more errors when the board rotated (p<0.05) (Fig 7).

## Discussion

This study compared spatial cognition of men and women in three tasks, two of them based on virtual reality technologies. Our analysis has shown that learning in the NWSBRT is far more complex than in the “walking” version of the same task (WSBRT). The stability of the viewpoint/starting position was associated with a more accurate performance in all the tasks. Furthermore, men outperformed women, committing less number of errors in both virtual tasks. However, no gender differences emerged in the spatial recall task (10/36 SRT).

Orientation in a context requires learning about the environmental features available. It is known that human beings use salient landmarks to organize the space of navigation. The position of these landmarks could be associated either with other landmarks or objects to form a spatial allocentric representation or with the individual’s position in a spatial egocentric representation. Free movement within the experimental environment, like that in the WSBRT, could help the adoption of viewpoint-dependent strategies. Thus, participants reach a target by finding a well-known position from which to retrieve the desired target. When the movement within the context is prevented, like in the NWSBRT, participants can only rely on a unique viewpoint and the flexible understanding of relationships between the landmarks available becomes essential.

NWSBRT was developed following the WSBRT, although some features were changed namely, the possibility of discovering the positions without displacement about the virtual room, and the possibility of catching a glimpse of the whole room. As our research has proven, participants made more errors in the non-walking (NWSBRT) than in the walking space virtual task (WSBRT). This is primarily due to the high number of mistakes in the rotating version of NWSBRT. Indeed, although three room walls are visible in the NWSBRT, participants made more errors in the identification of the rewarded positions. It should also be pointed out that NWSBRT does not provide the opportunity of changing the viewpoint in a trial and participants were to identify targets from a specific viewpoint (any of the four room walls). Previous research has reported that difficulty increases with the angular rotation of the training viewpoint [14, 15]. Conversely, in the rotating WSBRT only a partial set of stimuli were seen at a specific moment. Viewpoint changes as the participant explores the virtual room. Although this partial view of the room could be seen as a constraint, participants could, notwithstanding, adopt strategies based on snapshot captures of the targets and explore the context until they reach the viewpoint linked to a correct target identification.

In addition, stable versions were easier than rotating versions in both tasks. This is not surprising since rotation avoid egocentric task solutions. In the stable version of the NWSBRT participants can always perceive the room from the same viewpoint and can choose between various strategies, like associating landmarks with targets or even adopting egocentric solutions to remember rewarded positions (e.g. second box in the first line on the left). Although in the stable WSBRT participants always started from the same location they are free to move around the room. However, they visited the rewarded positions following almost the same sequence in the three and five rewards condition. This indicates that participants learn a path to reach the targets (route learning). Although forming allocentric strategies, like in the NWSBRT, is more flexible and they are acquired earlier [16], context features provide the opportunity to form egocentric strategies that could help to solve the task. As Iglói *et al*., (2009) [17] reported, in tasks with discrete targets like radial mazes, or even our task, allocentric and egocentric knowledge could be used from the very beginning.

Moreover, according to previous studies [7, 18], environment can also be processed differently depending on the nature of the space: near vs far space, both of which were assessed in this study by 10/36 SRT and the virtual tasks (NWSBRT and WSBRT), respectively. The assessment of performance in the near space (10/36 SRT) did not reveal any substantial group differences. Thus, cognitive demands of 10/36 SRT and virtual tasks (NWSBRT and WSBRT) differed. It is worthy to note that previous studies raised discrepancies between both types of tasks (i.e. classical visuospatial neuropsychological tests vs spatial memory test based on virtual reality technologies). This is probably due to the importance of a flexible representation of landmarks’ relationship in a spatial map that is not so demanded in other visuospatial tasks like the 10/36 SRT.

Special attention deserves the comparison between the stable and rotating versions of 10/36 SRT. Whereas participants improved their performance in the stable condition, board rotation clearly interfered with learning, as seen in Fig 7. Since there were not extramaze landmarks to facilitate orientation like in the NWSBRT and WSBRT, the only strategy available was the egocentric, i.e., to store in their memory the positions the pieces occupied on the board. Patterns described by pieces on the board could also help in the solution of the task. According to Wolbers and Wiener (2014) [19], if one’s own body is rotated in this kind of test, the egocentric distance and direction of the spatial targets would be affected. Hence, as participants do not have any external spatial cue each time the board is rotated a new spatial task is created.

Spatial memory in near and far spaces was previously compared with the use of another maze, the Corsi Block Tapping test in which participants had to remember a sequence of positions in the reaching and walking spaces [7]. Researchers adapted the traditional Corsi test, making participants move about to visit the various locations associated with the sequence to be remembered. The same procedure was also developed in a virtual environment [4], showing that performance in the virtual environment was more difficult. In addition, since targets can be reached in any order, tasks provide more freedom to unfold various type of strategies, although it affects difficulty [7]. Hence, Piccardi *et al*, (2014) [7] showed that remembering positions was easier when participants were not demanded to follow a sequence.

At the brain level, near and far spaces are represented in different brain structures or at least specific mechanisms as demonstrated by the dissociation found among patients with brain lesions [20]. In addition, fMRI studies proved that although there are common circuits involved in near and far space, some other brain structures are specific to one type of space [8].

Moreover, gender differences in spatial memory have been demonstrated in several species and tasks with males showing a more accurate performance than females [1, 12, 21, 22]. In this study, performance in the virtual reality-based tasks differed in men and women. Thus, men outperformed women in the stable condition in WSBRT and rotating NWSBR. Rotating NWSBRT involved an accurate spatial representation as well as a flexible understanding of landmarks relationships, since the context rotates in every trial. This involves an allocentric strategy, more common in men than in women. In addition, stable WSBRT provides the opportunity to follow several strategies. Regarding this, women generally prefer egocentric strategies and fix their attention in landmarks so as to orientate themselves [10, 23]. It has been proven that in unstable conditions many women take a concrete landmark as their reference point, starting their target search from this point, whereas men would reach targets directly from any start point [11]. Qualitative analysis of the sequence followed to reach targets in WSBRT showed that men and women repeated the same sequence in more than 6 trials. A possible explanation to this behavior is that both genders adopted the same strategy.

The rotating WSBRT previously disclosed gender dimorphism [2, 21, 24]. Nevertheless, no gender differences under this condition were found in this study. Differences in the experimental design could account for this discrepancy. Hence, in previous studies, each group of participants experienced a level of difficulty, thus revealing that men outperformed women when they searched for 5 rewards, but not in 3 and 7 difficulty levels. Now, the same participants received all treatments (3, 5 and 7 rewards). Accordingly, task experience could modulate gender differences [25].

It is necessary to point out that although videogame experience differed between men and women (see Table 1), differences emerged mainly in a task where joystick demands were reduced. However, since all participants performed the task within the time fixed, there seems to be no reason to think that videogame experience could account for this different performance. Note that in other versions of this task, gender differences emerged even when participants were passively guided through the context [26].

On the base of gender differences, hormone levels organize brain circuits and modulate neural functions [27, 28]. Hence, when hormone levels were modified during the early development, performance was affected [29]. In addition to this, the hormone level during the menstrual cycle could also contribute to performance in spatial tasks [30]. At brain level, different patterns of medial temporal lobe activity were associated with men and women spatial behavior [31].

In conclusion, this study has demonstrated that performance in walking and non-walking space can be measured with tasks with similar background and again, classical visuospatial tasks and new neuropsychological tasks based on virtual reality technologies provide different outcomes. This assessment revealed that tasks based on virtual reality technologies are very sensitive to small behavioral changes, like those associated with gender which manifests in both tasks. Since spatial memory abilities depend, to a large extent, on hippocampal integrity, the development of tasks and protocols focused on these cognitive skills could facilitate an early determination of brain disorders affecting these brain structures.
